# Thoracic Endovascular Aortic Repair for Retrograde Type A Aortic Intramural Hematoma

**DOI:** 10.3389/fcvm.2021.712524

**Published:** 2021-08-30

**Authors:** Gen Li, Xia Xu, Jun Li, Sizheng Xiong

**Affiliations:** ^1^Department of Cardiothoracic and Vascular Surgery, Tongji Hospital, Tongji Medical College, Huazhong University of Science and Technology, Wuhan, China; ^2^Key Laboratory of Organ Transplantation, Ministry of Education, Wuhan, China

**Keywords:** type A aortic intramural hematoma, penetrating atherosclerotic ulcer, aortic dissection, endovascular repair, aortic remodeling

## Abstract

**Objectives:** To evaluate the effects of thoracic endovascular aortic repair (TEVAR) in descending aorta for retrograde type A aortic intramural hematoma (re-TAIMH).

**Methods:** From January 2013 to September 2019, 65 consecutive patients diagnosed with re-TAIMH and treated by TEVAR were enrolled in this retrospective cohort study, of whom 44 patients presented with entry tear in descending aorta (Group A) and 21 with penetrating atherosclerotic ulcer (Group B). The clinical data, including baseline characteristics, adverse events, aortic remolding, and overall survival were reviewed.

**Results:** The mean age of all the patients was 52.0 ± 8.3 years, and 54 (83.1%) patients were men. The mean maximal ascending aortic diameter (MAAD) was 43.1 ± 5.4 mm, and the mean maximal ascending aortic hematoma thickness (MAAHT) was 9.6 ± 4.7 mm. TEVAR was performed under general anesthesia in 53 (81.5%) patients, while 12 (18.5%) patients were treated under local anesthesia. There were two deaths during hospitalization (one with rupture and another with multiple organ dysfunction syndrome), and overall survival at 1, 4, and 7 years for all 65 patients was 93.8, 92.0, and 87.4%, respectively. The MAAD and MAATH decreased significantly after TEVAR (*p* < 0.05) in the two groups, so did the mean descending aortic diameter at the pulmonary bifurcation level. Type I endoleak, dialysis, progression to type A aortic dissection, and enlargement in MAAHT and MAAD were more common complications, which occurred in four, three, two, and two patients, respectively.

**Conclusion:** Patients with retrograde TAIMH treated by TEVAR had a favorable prognosis including late survival and aortic remolding. However, some post-intervention complications were not negligible.

## Introduction

Aortic intramural hematoma (IMH), first described by Krukenberg (1), constitutes acute aortic syndrome (AAS) with penetrating atherosclerotic ulcer (PAU) and aortic dissection (AD) (1, 2). When hematoma involves ascending aorta, it is classified as Type A aortic intramural hematoma (TAIMH) (3). The cause of IMH is currently recognized as rupture of vasa-vasorum of aorta wall or intimal fracture induced by the progress of the atherosclerotic plaque (4). There is a certain degree of mutual transformation relationship among IMH, PAU, and AD, but the specific mechanism is not completely clear (5, 6). Generally, PAU in descending aorta can cause descending aortic hematoma, while some scholars also propose that the TAIMH could be induced by PAU located in descending aorta (7–9). Meantime, cases of TAIMH accompanied by descending aortic dissection are reported gradually (10–12). Therefore, the two rare and special entities, TAIMH with PAU or entry tear (intimal flap) in descending aorta, are named retrograde TAIMH (re-TAIMH) in current studies.

While how to treat uncomplicated TAIMH, emergency operation, or initial medical management is still in debate, thoracic endovascular aortic repair (TEVAR) in descending aorta for re-TAIMH is budding and applied successfully in some selected patients (8–12). The core idea of this technology is to block the PAU or entry tear, which may be the main culprit causing re-TAIMH. However, published studies are limited greatly in sample size. This study aims to evaluate the effects of TEVAR for re-TAIMH by analyzing the in-hospital and follow-up outcomes of 65 patients.

## Materials and Methods

### Study Design and Patients

From January 2013 to September 2019 at a single center, 65 consecutive patients diagnosed with re-TAIMH and treated by TEVAR were enrolled in this study, of whom 44 patients with TAIMH and entry tear in descending aorta were termed Group A, while 21 patients with TAIMH and PAU in descending aorta were termed Group B. The inclusion criteria were (a) patients with TAIMH, (b) patients with PAU or entry tear in descending aorta, and (c) patients treated by TEVAR or one-stop hybrid operation (TEVAR with aortic arch branch vessel bypass). The exclusion criteria were (a) patients with PAU or AD in ascending aorta and aortic arch, (b) patients with TAIMH and ulcer-like projection (ULP) in descending aorta, (c) patients treated by surgery, (d) patients with IMH that involves the arch without extension to the ascending aorta (13), and (e) patients with incomplete data including imaging and follow-up data.

This retrospective cohort study was approved by the ethics committee of the Tongji Hospital of Tongji Medical College of Huazhong University of Science and Technology, and individual patient consent was waived.

### Definition

**Figure 1:**
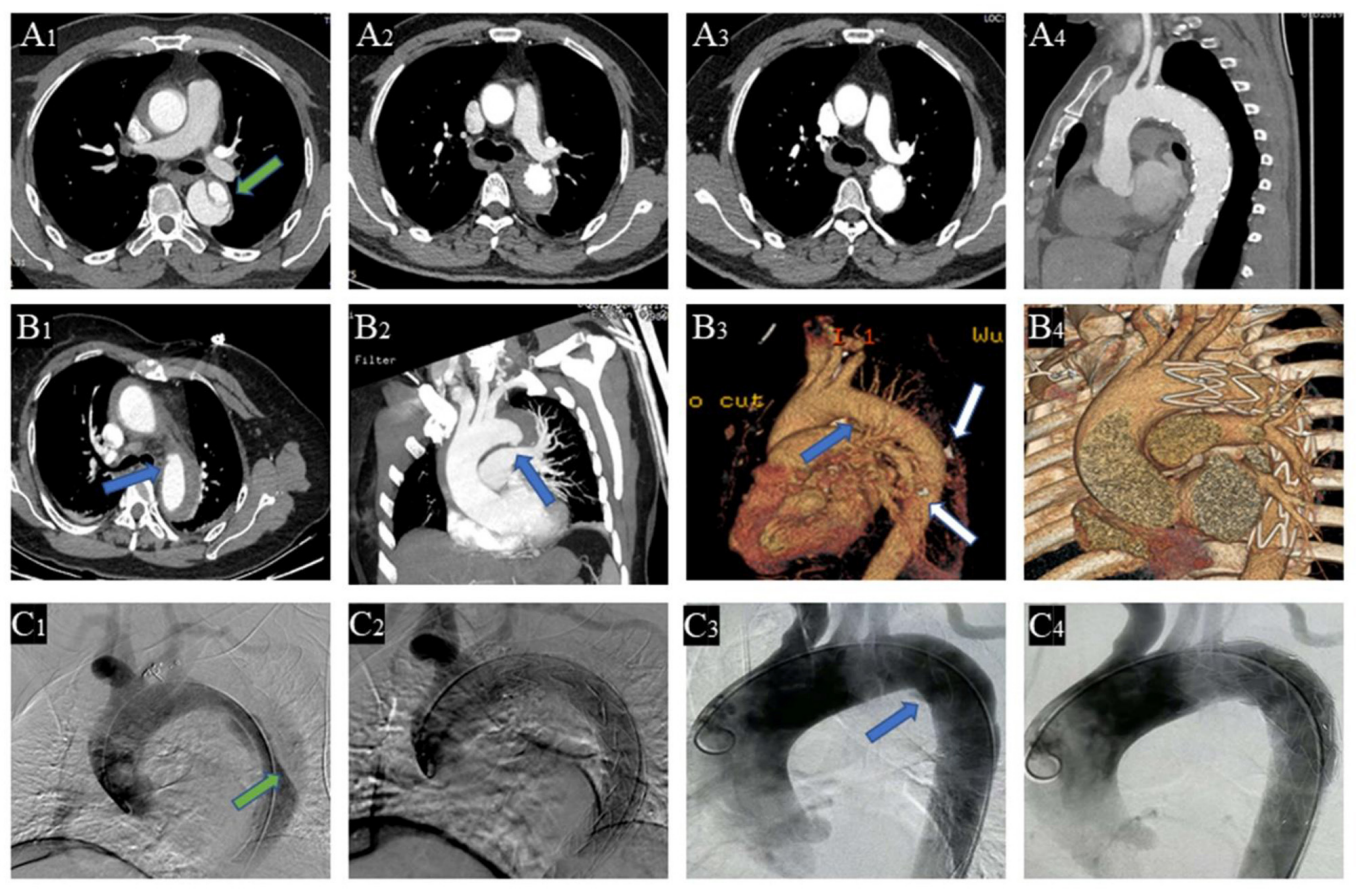
Contrast-enhanced computed tomography angiography (CTA) of the patients with retrograde type A aortic intramural hematoma (TAIMH). A patient with entry tear (green arrow) in descending aorta was treated by thoracic endovascular aortic repair (TEVAR), and the image was collected at admission **(A**_**1**_**)**, 2 weeks after TEVAR **(A**_**2**_**)**, and 3 months after TEVAR **(A**_**3,4**_**)**, respectively. Another patient with penetrating atherosclerotic ulcer (PAU, blue arrow) and calcification plaque (white arrow) at admission **(B**_**1−3**_**)** and 2 weeks after TEVAR **(B**_**4**_**)**, respectively. Intraoperative angiography of the two patients with retrograde TAIMH before (**C**_**1**_, entry tear, green arrow; **C**_**3**_, PAU, blue arrow) and after implanting stent grafts (**C**_**2**_, entry tear was blocked; **C**_**4**_, PAU was blocked).

Contrast-enhanced computed tomography angiogram (CTA) was used to diagnose different entities and measure parameters. TAIMH is defined as hemorrhage consisting of a circular or crescentic thickening around the ascending aorta, and there is no blood flow between the lumen and the aortic wall (14). PAU is defined as an aortic atherosclerotic lesion in the internal elastic lamina that penetrates the media and is often accompanied by aortic calcification plaque (6, 14). Re-TAIMH was defined as TAIMH with PAU or entry tear in descending aorta (Figure 1). Meantime, the PAU will cause descending aortic hematoma, and the entry tear is a defect of the intimal flap in type B AD separating the true lumen (TL) and the false lumen (FL).

The CTA parameters, referring to the maximal ascending aortic diameter (MAAD), the maximal ascending aortic hematoma thickness (MAAHT), the descending aortic diameter (DAD), and the ratio of TL diameter to DAD, were measured at near pulmonary artery bifurcation level. In this article, TEVAR refers specifically to endovascular repair in the descending aorta to avoid confusion with ascending aortic endovascular repair.

### Management

The clinical management approach of TEVAR for patients with re-TAIMH is described below. All the patients diagnosed with re-TAIMH by CTA on admission were admitted to the intensive care unit with careful monitor, and they would receive medical treatment to control blood pressure, heart rate, and pain. The following conditions were considered contraindications of TEVAR for re-TAIMH: (a) patients with hemodynamic instability, (b) patients with valve disease or coronary artery disease or other diseases that needed additional surgical or interventional treatment besides TEVAR or hybrid operation, (c) patients with massive pericardial effusion (cardiac tamponade) or aortic arch branch ischemia, and (d) patients with no appropriate access route for the stent graft. In patients without these conditions, TEVAR was recommended as a preferred treatment for some of them with high risks of surgery including old age and severe comorbidities, which were assessed by the surgeons and anesthetists, and it was recommended as an alternative treatment for other patients without the risks. Eventually, TEVAR was performed according to the medical suggestions and with the consent of the patients and their families.

The timing of TEVAR depended on a complex of events including malperfusion, persistent pain, uncontrolled hypertension, and progression on CTA reexamination before intervention. During hospitalization, the patients were normally assessed by CTA every week after admission unless emergency and 1–2 weeks after intervention as well. They were usually discharged 15 days after intervention under the conditions of stable vital signs and confirmed improvement on imaging.

### Follow-Up

Patients were followed up by clinic reexamination, telephones, and social apps. The mean follow-up time was 46.6 ± 21.2 months. After discharge, CTA was performed at 3 months, 6 months, and 1 year after TEVAR in our center and annually thereafter in the local hospital near the patients for convenience and low costs. Eventually, CTA records of the patients from our center at 1 year after TEVAR were analyzed.

### TEVAR Procedures

The thoracic stent grafts implanted were Valiant (Medtronic, Minneapolis, MN, USA), Relay (Bolton Medical, Sunrise, FL, USA), and Hercules (Micropart, Shanghai, CN, USA). All TEVAR procedures were performed in the hybrid operating room with femoral cutdown access under the management of a multidisciplinary team. Normally, TEVAR was performed under general anesthesia, while patients with difficult airway or obstructive sleep apnea or severe chronic obstructive pulmonary disease were treated under local anesthesia with intravenous opiates. TEVAR was aimed at excluding the PAU or entry tear in the descending aorta. When the proximal landing zone affected the blood flow of the aortic arch branch, one or combined adjunctive procedures including aortic arch branch vessel bypass, handmade fenestrated stent graft, covered left subclavian artery (LSCA) with no revascularization were applied. The handmade fenestrated stent graft was made according to the preoperative CTA and the aortogram during operation, by which we marked the stent via measuring the distance among the aortic arch branch vessels. Then, we created fenestrations on Valiant or Relay devices by the high-temperature cauteries (Bovie, Symmetry Surgical, TN, USA). The stent graft was oversized to the mean aortic diameter (average of the maximum and minimum values) of proximal landing zone by 5–10%. Blood pressure was controlled in the whole procedure with a systolic pressure of 70–90 mmHg.

### Statistical Analysis

Categorical variables were summarized as number and percent, and continuous variables were described using mean ± SD or median (first quartile, third quartile). We assessed the difference between Group A and Group B using independent sample *t*-test or Mann–Whitney *U*-test depending on normal or non-normal distribution for continuous variables, and chi square (χ^2^) or Fisher's exact tests were used for categorical variables as appropriate. The changes in CTA parameters before and after TEVAR were analyzed using a paired *t*-test. Survival curve was plotted using the Kaplan–Meier method and examined using the log-rank test. The statistical threshold for significance for all analyses was *p* = 0.05. Data analyses were performed using R software (version 3.6.1; R Foundation, Vienna, Austria).

## Results

### Clinical Characteristics on Admission

A total of 65 patients were identified with re-TAIMH. Their mean age was 52.0 ± 8.3 years, and 54 (83.1%) patients were men (Table 1). The most common symptom was chest pain (87.7%), and the most common comorbidity was hypertension (84.6%). There were seven (10.8%) patients with abnormal liver function on admission. The mean estimated glomerular filtration rate was 75.1 ± 27.5 ml/min/1.73 m^2^, and the median D-dimer level was 6.7 (3.2, 14.3) μg/ml. There was no statistical difference in the baseline characteristics between Group A and Group B except that the D-dimer level in Group A was higher than that in Group B (median, 12.1 vs. 4.3 μg/ml, *p* = 0.008, Table 1).

**Table 1:** Clinical characteristics in patients with entry tear (Group A) and penetrating atherosclerotic ulcer (Group B) on admission.

	**Total *N* = 65**	**Group A *N* = 44**	**Group B *N* = 21**	***p-*value**
Age, years	52.0 ± 8.3	52.1 ± 9.3	51.6 ± 5.9	0.825
Sex, male	54 (83.1%)	39 (88.6%)	15 (71.4%)	0.154
Smoking history	16 (24.6%)	12 (27.3%)	4 (19.0%)	0.472
Symptom				0.106
Chest and back pain	57 (87.7%)	39 (88.6%)	18 (85.7%)	
Abdominal pain	6 (9.2%)	5 (11.4%)	1 (4.8%)	
Neurological deficit	2 (3.1%)	0 (0%)	2 (9.5%)	
Comorbidities				
Hypertension	55 (84.6%)	40 (90.9%)	15 (71.4%)	0.065
Dyslipidemia	19 (29.2%)	12 (27.3%)	7 (33.3%)	0.615
Diabetes mellitus	3 (4.6%)	2 (4.5%)	1 (4.8%)	>0.999
COPD	10 (15.4%)	7 (15.9%)	3 (14.3%)	>0.999
Cerebrovascular disease	6 (9.2%)	4 (9.1%)	2 (9.5%)	>0.999
Coronary artery disease	8 (12.3%)	5 (11.4%)	3 (14.3%)	0.706
Aberrant right subclavian artery	1 (1.5%)	0 (0%)	1 (4.8%)	0.323
Laboratory findings				
Abnormal liver function^*^	7 (10.8%)	6 (13.6%)	1 (4.8%)	0.413
eGFR, ml/min/1.73 m^2^	75.1 ± 27.5	71.7 ± 28.9	82.1 ± 23.5	0.157
D-dimer, μg/ml	6.7 (3.2, 14.3)	12.1 (4.3,19.6)	4.3 (1.4, 8.3)	0.008
LVEF, %	52.8 ± 5.8	53.3 ± 5.5	50.3 ± 7.2	0.163

*Categorical variables were presented as number (%). Continuous variables were presented as mean ± standard deviation or median (first quartile, third quartile)*.

*COPD, chronic obstructive pulmonary disease; eGFR, estimated glomerular filtration rate; LVEF, left ventricular ejection fraction*.

* *Aminotransferase or alanine aminotransferase beyond upper limit of the normal value*.

### CTA Parameters on Admission and Intervention Details

The number of patients with dissection extending below the diaphragm was 36 (81.8%) in Group A, while the number of patients with IMH extending below the diaphragm was 16 (76.2%) in Group B. The mean MAAD was 43.1 ± 5.4 mm, and the mean MAAHT was 9.6 ± 4.7 mm in all patients. Details about the CTA parameters on admission are shown in Table 2.

**Table 2:** Contrast-enhanced computed tomography angiogram parameters in patients with entry tear (Group A) and penetrating atherosclerotic ulcer (Group B) on admission.

	**Total *N* = 65**	**Group A *N* = 44**	**Group B *N* = 21**	***p-*value**
Extent of dissection/IMH in descending aorta				0.742
Above the diaphragm	13 (20.0%)	8 (18.2%)	5 (23.8%)	
Below the diaphragm	52 (80.0%)	36 (81.8%)	16 (76.2%)	
Maximal ascending aortic diameter, mm	43.1 ± 5.4	42.4 ± 5.5	44.6 ± 4.9	0.127
Maximal ascending aortic hematoma thickness, mm	9.6 ± 4.7	9.4 ± 5.1	9.9 ± 4.1	0.821
Distance between LSCA and entry tear/PAU, mm	29.1 ± 15.6	29.4 ± 17.5	28.3 ± 10.7	0.795
Pericardial effusion^*^	16 (24.6%)	10 (22.7%)	6 (28.6%)	0.609
Pleural effusion	32 (49.2%)	23 (52.3%)	9 (42.9%)	0.478

*Categorical variables were presented as number (%). Continuous variables were presented as mean ± standard deviation*.

*IMH, aortic intramural hematoma; LSCA, left subclavian artery; PAU, penetrating atherosclerotic ulcer*.

* *Small amount without hemodynamic instability*.

The median interval from admission to TEVAR was 2.0 (1.0, 3.0) days, and patients in Group B had a longer interval than patients in Group A (median, 3.0 vs. 1.0 days, *p* = 0.001, Table 3). TEVAR was performed under general anesthesia in 53 (81.5%) patients, while 12 (18.5%) patients were treated under local anesthesia. The number of patients with proximal landing zones in Zones 1–3 was 4 (6.2%), 40 (61.5%), and 21 (32.3%), respectively. The mean aortic diameters of the proximal and distal landing zone were 31.4 ± 3.4 mm and 25.8 ± 3.4 mm, respectively (Table 3). One patient underwent axillary to axillary and LCCA bypass, and the LSCA was intentionally covered in 10 (15.4%) patients with non-revascularization. The handmade fenestrated stent grafts were applied in seven (10.8%) patients. At the end of the intervention, six (9.2%) patients received pleural drainage with a center venous catheter for moderate to large pleural effusion found in preoperation CTA.

**Table 3:** Intervention details in patients with entry tear (Group A) and penetrating atherosclerotic ulcer (Group B).

	**Total *N* = 65**	**Group A *N* = 44**	**Group B *N* = 21**	***p-*value**
Time from admission to TEVAR, days	2.0 (1.0, 3.0)	1.0 (1.0, 2.0)	3.0 (2.0, 8.5)	0.001
Anesthesia				0.180
General anesthesia	53 (81.5%)	38 (86.4%)	15 (71.4%)	
Local anesthesia	12 (18.5%)	6 (13.6%)	6 (28.6%)	
Number of stent grafts				0.363
1	17 (26.2%)	10 (22.7%)	7 (33.3%)	
2	48 (73.8%)	34 (77.3%)	14 (66.7%)	
Proximal landing zone				0.908
1	4 (6.2%)	3 (6.8%)	1 (4.8%)	
2	40 (61.5%)	26 (59.1%)	14 (66.7%)	
3	21 (32.3%)	15 (34.1%)	6 (28.6%)	
Aortic diameters of proximal landing zone, mm	31.4 ±3.4	30.4 ± 3.0	33.5 ± 3.4	<0.001
Aortic diameters of distal landing zone, mm	25.8 ± 3.4	24.8 ± 3.2	28.0 ± 2.8	<0.001
Adjunctive procedures				
Axillary to axillary and LCCA bypass	1 (1.5%)	1 (2.3%)	0 (0%)	>0.999
Covered LSCA, non-revascularization	10 (15.4%)	7 (15.9%)	3 (14.3%)	>0.999
Handmade fenestrated stent graft	7 (10.8%)	4 (9.1%)	3 (14.3%)	0.672
Pleural drainage after TEVAR	6 (9.2%)	4 (9.1%)	2 (9.5%)	>0.999

*Categorical variables were presented as number (%). Continuous variables were presented as mean ± standard deviation or median (first quartile, third quartile)*.

*TEVAR, thoracic endovascular aortic repair; LCCA, left common carotid artery; LSCA, left subclavian artery*.

### Outcomes and Survival

Patients with post-operation complications are listed in Table 4. Two patients in Group A died during hospitalization. One died of aortic rupture 3 days after TEVAR, which was confirmed by bedside transthoracic echocardiography. Another died of multiple organ dysfunction syndrome secondary to pulmonary infection 24 days after TEVAR.

**Table 4:** Outcomes after endovascular repair in patients with entry tear (Group A) and penetrating atherosclerotic ulcer (Group B).

**No**.	**Group**	**Age**	**Sex**	**Outcomes (in-hospital)**	**In-hospital mortality**	**Outcomes (after discharge)**	**Late mortality**
1	A	45	M	Rupture	Yes	None	None
2	A	49	M	Pulmonary infection, dialysis, and MODS	Yes	None	None
3	A	46	M	Type I endoleak	No	Endoleak disappeared	No
4	A	62	M	Dialysis	No	Renal function recovered	No
5	A	65	F	None	No	Progression to type A AD and rupture	Yes
6	A	58	M	None	No	Cerebral hemorrhage	Yes
7	A	55	M	None	No	Type I endoleak	No
8	A	43	M	None	No	Type I endoleak	No
9	A	42	M	None	No	Re-intervention	No
10	B	53	M	SINE, MAAHT, and MAAD enlargement	No	IMH absorption MAAD decreased SINE stabilized	No
11	B	49	M	MAAHT and MAAD enlargement	No	IMH absorption MAAD decreased	No
12	B	50	M	Type I endoleak	No	Endoleak existed	No
13	B	66	M	Dialysis	No	Renal function recovered	No
14	B	56	F	None	No	Progression to type A AD and rupture	Yes
15	B	54	M	None	No	Sudden death	Yes

*M, male; F, female; MODS, multiple organ dysfunction syndrome; AD, aortic dissection; SINE, stent graft-induced new entry; MAAHT, maximal ascending aortic hematoma thickness; MAAD, maximal ascending aortic diameter*.

**Figure 2:**
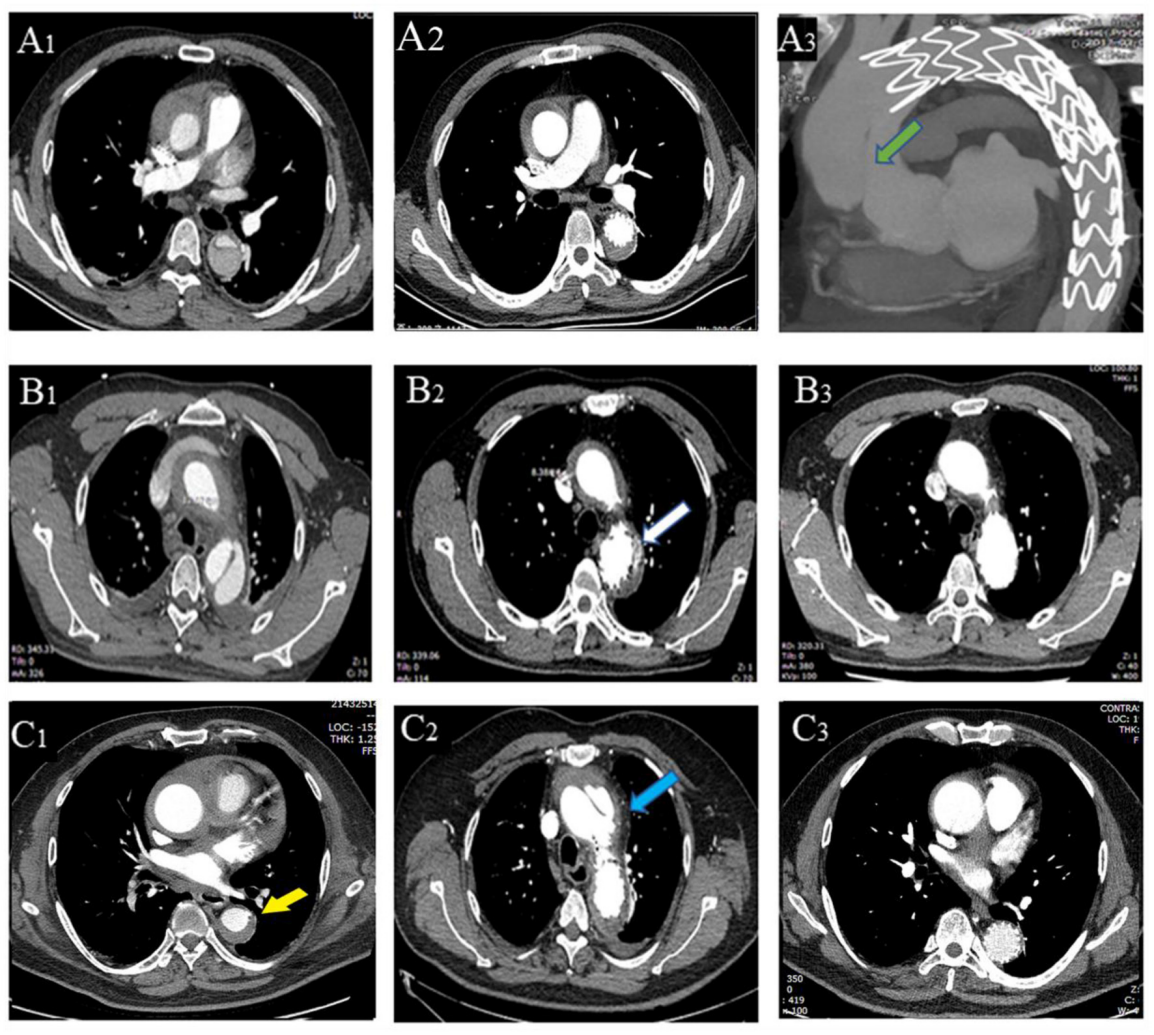
Thoracic endovascular aortic repair (TEVAR)-related complications. A patient with entry tear on admission **(A**_**1**_**)**, 2 weeks after TEVAR **(A**_**2**_**)**, and had type A aortic dissection 4 months **(A**_**3**_**)** after TEVAR (new entry tear in ascending aorta, green arrow). (**B**_**1–3**_) A patient with entry tear had type I endoleak (**B**_**2**_, white arrow) 2 weeks after TEVAR, which disappeared 6 months later **(B**_**3**_**)**. A patient with penetrating atherosclerotic ulcer (PAU, yellow arrow, **C**_**1**_) had aortic arch dissection (blue arrow, **C**_**2**_) induced by proximal portion of stent graft with hematoma thickening 2 weeks after TEVAR, and the dissection was confined within the aortic arch with hematoma disappearing **(C**_**3**_**)**.

At the first reexamination 14 days after TEVAR, one patient in Group A had type I endoleak (Figure 2). In Group B, one patient had aortic arch dissection induced by the proximal portion of stent graft and enlargement in MAAHT and MAAD (Figure 2), one had type I endoleak, and one had MAAHT and MAAD enlargement. All these patients chose to receive medical treatment and were alive, and changes in these complications during follow-up are shown in Table 4.

After discharge, there were two deaths in each group (Table 4). One patient in Group A died of progression to type A AD and rupture 4 months after TEVAR and refused surgical treatment (Figure 2). Another one died of cerebral hemorrhage 6 years after TEVAR. In Group B, a patient developed type A AD and died of rupture on the way to referral, and another one encountered sudden death 2.7 years after TEVAR. In Group A, two patients had type I endoleak after discharge, and one patient was treated by endovascular repair of abdominal aortic dissection with pseudoaneurysm (Table 4).

**Figure 3:**
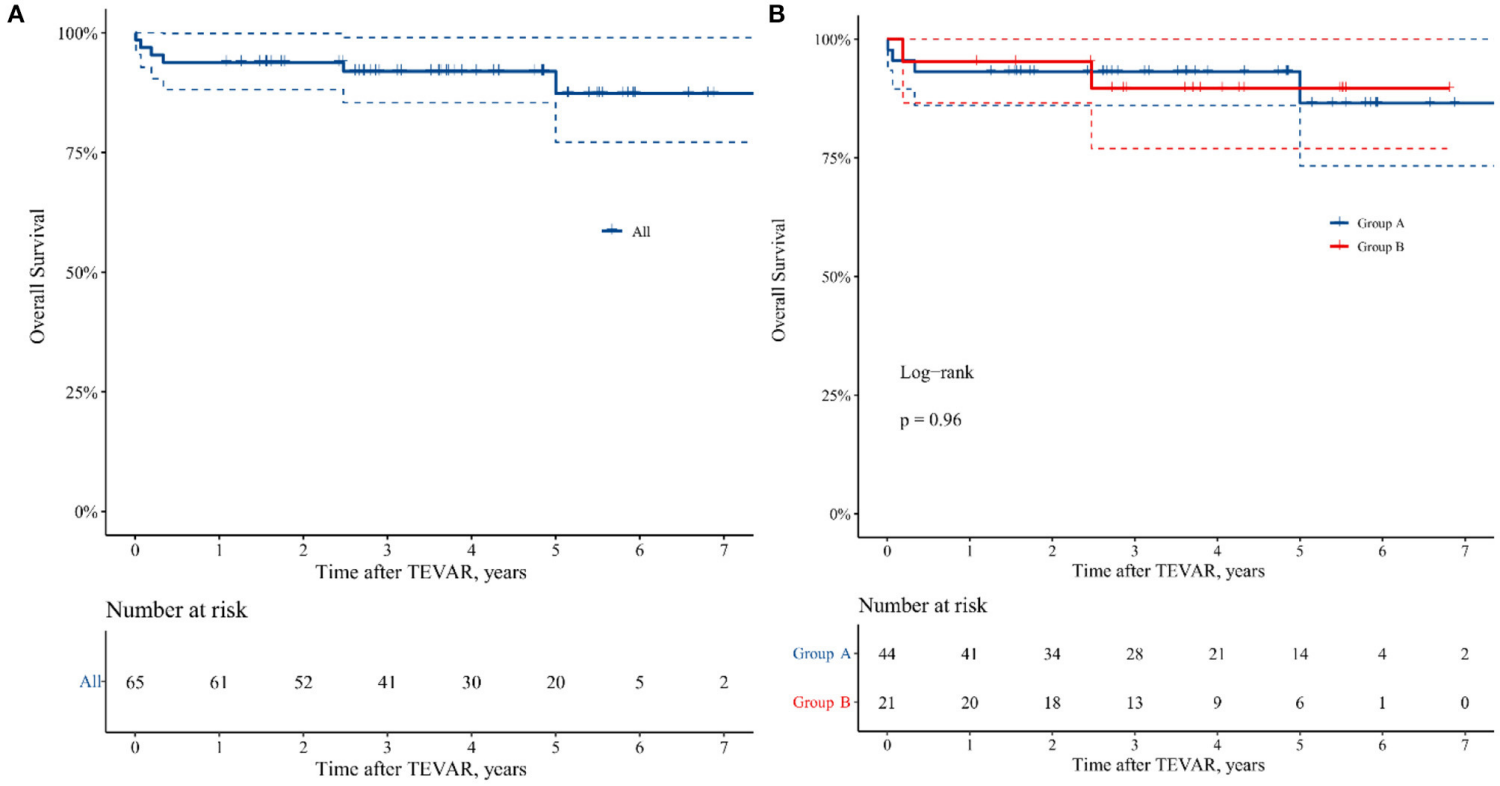
Kaplan–Meier curve of 7 years freedom from all causes mortality in the patients undergoing TEVAR. **(A)** All the patients. **(B)** Group A: patients with TAIMH and entry tear in descending aorta, and Group B: patients with TAIMH and penetrating atherosclerotic ulcer (PAU) in descending aorta.

The overall survival at 1, 4, and 7 years for all 65 patients was 93.8, 92.0, and 87.4%, respectively (Figure 3), and there was no statistical difference in late-term survival between the two groups (*p* = 0.963, Figure 3).

### Aortic Remodeling 1 Year After TEVAR

**Figure 4:**
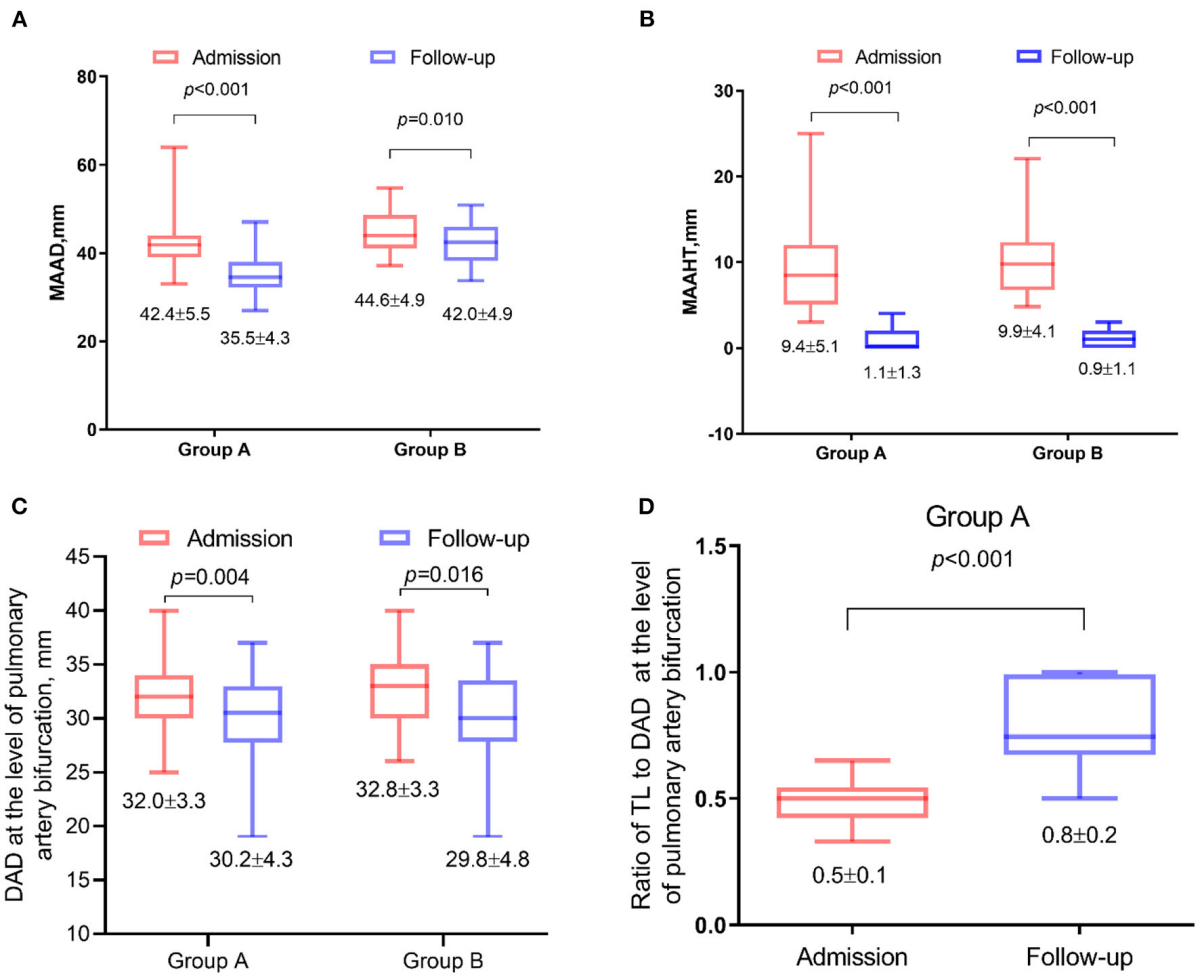
Changes in contrast-enhanced computed tomography angiography (CTA) parameters 1 year after thoracic endovascular aortic repair (TEVAR). Data were plotted as box-and-whiskers plots. The upper and lower borders of the box represented the upper and lower quartiles. The middle horizontal line represented the median. The upper and lower whiskers represent the maximum and minimum values. The values of mean and standard deviation were also showed below. **(A)** Comparison of the maximal ascending aortic diameter (MAAD). **(B)** Maximal ascending aortic hematoma thickness (MAAHT). **(C)** The mean descending aortic diameter at the pulmonary bifurcation level. **(D)** The ratio of true lumen (TL) to descending aortic diameter at the pulmonary bifurcation level.

The MAAD and MAATH all decreased significantly in the two groups (*p* < 0.05, Figure 4), and 52.5% of patients achieved IMH total absorption in ascending aorta. The mean descending aortic diameter at the pulmonary bifurcation level also all decreased significantly (*p* < 0.05, Figure 4). In Group A, the ratio of TL to descending aortic diameter at the pulmonary bifurcation level increased significantly after TEVAR (*p* < 0.001, Figure 4).

## Discussion

To our knowledge, this was the current study focusing on re-TAIMH with the largest sample size. It showed that patients with re-TAIMH treated by TEVAR had a favorable prognosis including late survival and aortic remolding. Meantime, some adverse events that had not been reported yet developed with the cases increasing, which was not negligible.

Although the consensus had not been reached on the treatment strategy of TAIMH in different areas, surgery was suggested when TAIMH was accompanied by PAU, which was a risk factor of TAIMH progression (14, 15). However, the idea about endovascular repair of re-TAIMH did not occur in a vacuum. TEVAR was recommended to treat complicated type B AAS for a long time because of its good results, and previous studies showed that TEVAR for type A AD with entry tear in descending aorta seemed promising, which provided a hypothesis that blocking PAU or entry tear, the primary lesion, could prevent progression of re-TAIMH (16–18). Although there were no consecutive imaging studies that confirmed the process that the entry tear or PAU in descending aorta induced re-TAIMH, previous studies based on this hypothesis dealing solely with descending aortic lesions by TEVAR had achieved good results with the reduced risk of rupture or death during hospitalization and the favorable remolding of the entire aorta during follow-up, which in turn supported this hypothesis (7, 10, 12, 19, 20). The most serious complication of IMH was progression to AD and/or rupture, and when the ascending aorta was involved, the mortality was greatly increased (11, 14). In group A, we prevented transformation to type A aortic dissection by sealing the entry tear and reduce the blood pressure within the FL to improve malperfusion. While in Group B, we prevented first the aortic rupture or progression to AD and reduced the aortic wall thickening (19).

It was reported that the early mortality rate of 168 patients with TAIMH treated surgically was 10.1%, and the 5-year survival rate of 83 patients with TAIMH was 65 ± 22% in a clinical review (21). A recent cohort reported that the 30-day mortality of 101 patients with TAIMH who underwent surgery was 11.9% (22). By contrast, the effect of TEVAR for re-TAIMH was more striking. In previous studies with a total number of 37 patients, there was no in-hospital mortality, and two patients died in the follow-up of 24 months, of which a cohort including 18 patients achieved 100% overall survival rate and favorable remolding during 28.7 ± 18.9 months (7, 8, 10, 19, 23). However, the inadequacy of the sample size reduced the persuasiveness of these studies. Our study with relatively more cases showed the same satisfactory trend in patients with re-TAIMH treated by TEVAR. Meantime, there were some differences.

Re-TAIMH was currently a relatively obscure concept that TAIMH accompanied by PAU or ULP in descending aorta or type B AD was all included (7–10, 12). In recent years, the small tear or rupture of the intima was found in CTA and was confirmed during the operation within some patients with IMH (24–26). Therefore, IMH was also called thrombosed-type AD (27). Meantime, it suggested that aortic atherosclerotic plaque rupture might be identified as the cause of IMH, which could extend retrogradely toward the ascending aorta (7). However, researchers could be easily confused by PAU and ULP. The tremendous advancement of imaging technology could distinguish them (28). ULP was characterized by a broadly communicating saccular area, and its interface with the surrounding hematoma was smooth. By contrast, PAU was usually accompanied by atheromatous changes, and associated IMH has an undulating interface of low-attenuation ulcerated plaque and thrombus with contrast enhancement (28). ULP might appear within the first days or several months after the acute onset of symptoms along with the evolution of IMH, which was believed to be a new intimal disruption following IMH (3, 29, 30). Although these associations had not been systematically confirmed in the pathology, we excluded patients with ULP in this study because our hypothesis was to handle the “initiating factors” rather than the likely secondary lesion by endovascular repair.

In contrast between the patients in the two groups, there were few differences with statistical significance. Patients with entry tear were more likely to have a higher level of D-dimer than patients with PAU, which might be attributed to the larger breach in intima and communication between TL and FL. The aortic diameters of proximal and distal landing zone were larger in patients with PAU because of the relatively larger MAAD in patients with PAU on admission and the different number of stents implanted. In addition, AD usually caused malperfusion of internal organs or lower limbs, which seldom developed in PAU or IMH. Thus, patients with PAU would be observed with more time before TEVAR was performed than patients with entry tear. These differences might be another form of unity because the entry tear characterized by larger size caused classical AD in descending aorta and thrombosed-type AD in ascending aorta, while PAU characterized by smaller size caused entire thrombosed-type AD.

As for the adverse event after TEVAR, some complications including endoleak, dialysis, and rupture with low incidence rate had been also reported in similar studies previously (10, 16). However, new entry tear developing in ascending aorta or MAAHT and MAAD enlargement was rare in published research, which seemed like a contradiction under the situation that the primary lesion was blocked. But if there are other tears in the ascending aorta or aortic arch that is not protected by the stent, the disease could worsen because of the increasing pressure of the proximal IMH (20). This possibility would constrain the application of TEVAR for re-TAIMH, and indeed, the thicker ascending aortic hematoma would interfere with the evaluation of CTA images and was also found to be a risk factor for the prognosis in patients with TAIMH (14). Therefore, selected patients, appropriate timing, and a skilled team would always be a threshold for endovascular repair of re-TAIMH before improved imaging test could identify the whole lesions thoroughly.

It is worth mentioning that some patients considered unsuitable for general anesthesia by the anesthesiologists were treated under local anesthesia. During the process, careful operation, sufficient pain control, and close communication between the anesthesiologist and the surgeon were key points to obtain a good result (31). When the proximal landing zone affected the blood flow of the aortic arch branch, different adjunctive procedures including branched stent grafts, chimney stent, hybrid operation, LSCA embolization, and fenestrated stent graft would work out the dilemma. However, it was difficult to judge which approach was the best one and usually necessary to make a suitable plan according to the individual character of the patient.

This study had some limitations. First, it was a single-center retrospective study with a limited number of patients. Second, CTA reexamination after TEVAR kept time synchronization only at near 1 year, which prevented us from evaluating long-term aortic remolding, although the patients achieved favorable remolding in the early stage. Third, no health landing zone existed in the intervention, and when we avoided excessive damage to the aortic wall, type I endoleak developed in four patients. Fourth, LSCA was covered with non-revascularization in patients without dominant left vertebral artery when the additional proximal landing zone was needed in emergency TEVAR. Although neurological complications did not develop, the associated risk existed. Last, although we had recommended aggressive treatment, most patients with adverse events chose to wait and see and refused surgical treatment or reintervention.

## Conclusion

TEVAR is a feasible alternative approach for the treatment of re-TAIMH. The favorable prognosis including mid-term survival and aortic remolding at an early stage could be achieved in patients with re-TAIMH treated by TEVAR. However, this technology should be performed with appropriate indication, a skilled team, and excellent management at present because post-intervention complications like new entry tear in ascending aorta or MAAHT and MAAD enlargement might develop with unclear mechanism, although the probability was small. Besides, the follow-up of imaging tests should be rigorously implemented to find progression timely and gain time for remedies.

## Data Availability Statement

The raw data supporting the conclusions of this article will be made available by the authors, without undue reservation.

## Ethics Statement

The studies involving human participants were reviewed and approved by Ethics Committee of the Tongji Hospital of Tongji Medical College of Huazhong University of Science and Technology. Written informed consent for participation was not required for this study in accordance with the national legislation and the institutional requirements.

## Author Contributions

SX was involved in the conceptualization and study design, and completed the writing review and editing. JL contributed to the project administration and supervision. XX contributed to the review of computed tomography data. GL and XX collected the data. GL performed the statistical analysis and wrote the manuscript draft. All authors contributed to the article and approved the submitted version.

## Conflict of Interest

The authors declare that the research was conducted in the absence of any commercial or financial relationships that could be construed as a potential conflict of interest.

## Publisher's Note

All claims expressed in this article are solely those of the authors and do not necessarily represent those of their affiliated organizations, or those of the publisher, the editors and the reviewers. Any product that may be evaluated in this article, or claim that may be made by its manufacturer, is not guaranteed or endorsed by the publisher.

## Abbreviations

IMH: aortic intramural hematoma
AAS: acute aortic syndrome
PAU: penetrating atherosclerotic ulcer
AD: aortic dissection
TAIMH: type A aortic intramural hematoma
ULP: ulcer-like projection
Re-TAIMH: retrograde type A aortic intramural hematoma
TEVAR: thoracic endovascular aortic repair
CTA: contrast-enhanced computed tomography angiogram
TL: true lumen
FL: false lumen
MAAD: maximal ascending aortic diameter
MAAHT: maximal ascending aortic hematoma thickness
DAD: descending aortic diameter
LSCA: left subclavian artery.
